# Integrative Transcriptomics and Proteomics Analyses to Reveal the Developmental Regulation of *Metorchis orientalis*: A Neglected Trematode With Potential Carcinogenic Implications

**DOI:** 10.3389/fcimb.2021.783662

**Published:** 2021-12-02

**Authors:** Jun-Feng Gao, Qing-Bo Lv, Rui-Feng Mao, Yun-Yi Sun, Ying-Yu Chen, Yang-Yuan Qiu, Qiao-Cheng Chang, Chun-Ren Wang

**Affiliations:** College of Animal Science and Veterinary Medicine, Heilongjiang Bayi Agricultural University, Daqing, China

**Keywords:** *Metorchis orientalis*, transcriptome, proteome, adult stage, metacercariae stage, differentially expressed genes

## Abstract

*Metorchis orientalis* is a neglected zoonotic parasite of the gallbladder and bile duct of poultry, mammals, and humans. It has been widely reported in Asian, including China, Japanese, and Korea, where it is a potential threat to public health. Despite its significance as an animal and human pathogen, there are few published transcriptomic and proteomics data available. Transcriptome Illumina RNA sequencing and label-free protein quantification were performed to compare the gene and protein expression of adult and metacercariae-stage *M. orientalis*, resulting in 100,234 unigenes and 3,530 proteins. Of these, 13,823 differentially expressed genes and 1,445 differentially expressed proteins were identified in adult versus metacercariae. In total, 570 genes were differentially expressed consistent with the mRNA and protein level in the adult versus metacercariae stage. Differential gene transcription analyses revealed 34,228 genes to be expressed in both stages, whereas 66,006 genes showed stage-specific expression. Compared with adults, the metacercariae stage was highly transcriptional. GO and KEGG analyses based on transcriptome and proteome revealed numerous up-regulated genes in adult *M. orientalis* related to microtubule-based processes, microtubule motor activity, and nucleocytoplasmic transport. The up-regulated genes in metacercariae *M. orientalis* were mainly related to transmembrane receptor protein serine/threonine kinase activity, transmembrane receptor protein serine/threonine kinase signaling pathway. Transcriptome and proteome comparative analyses showed numerous up-regulated genes in adult stage were mainly enriched in actin filament capping, spectrin, and glucose metabolic process, while up-regulated genes in metacercariae stage were mainly related to cilium assembly, cilium movement, and motile cilium. These results highlight changes in protein and gene functions during the development of metacercariae into adults, and provided evidence for the mechanisms involved in morphological and metabolic changes at both the protein and gene levels. Interestingly, many genes had been proved associated with liver fibrosis and carcinogenic factors were identified highly expressed in adult *M. orientalis*, which suggests that *M. orientalis* is a neglected trematode with potential carcinogenic implications. These data provide attractive targets for the development of therapeutic or diagnostic interventions for controlling *M. orientalis*.

## Introduction

The Opisthorchiidae is a large family of trematodes causing diseases with significant socioeconomic impacts in humans and animals in Asia and Europe, with more than 10 million people affected and ~680 million people estimated to be at risk of infection (Saijuntha et al., 2021). Opisthorchiidae flukes inhabit the biliary tract of the host, causing chronic diseases, including cholangitis, cholecystitis, cholelithiasis, and cholangiocarcinoma. Despite their significance, many of them have been neglected in terms of research and their control. *Metorchis orientalis* is a freshwater fluke and one such neglected member of the Opisthorchiidae. It mainly inhabits the gallbladder and bile duct of poultry and mammals, including humans. *M. orientalis* is endemic predominantly in regions of Korea and China (Qiu et al., 2017; Zhan et al., 2017; Sohn et al., 2021), where it has a wide geographical distribution across 19 provinces in China, thus representing a significant socioeconomic burden (Gao et al., 2018).

The life cycle of *M. orientalis* is very similar to that of *Clonorchis sinensis*. It includes two intermediate aquatic hosts (aquatic snails and freshwater fish) and a definitive host (piscivorous poultry and mammals). The first intermediate host (aquatic snail) is infected *via* consumption of embryonated eggs released with the feces of the definitive host. After asexual development in the snails, the cercarial stage is released and swims in search of its second intermediate hosts (freshwater fish). It then penetrates the skin of the fish and encysts as a metacercariae. Metacercariae are the infective stage of the fluke and its definitive hosts (poultry and mammals) become infected *via* the consumption of raw or undercooked infected freshwater fish. The metacercariae excyst in the duodenum and migrate into the bile duct, where they develop into adult flukes. The eggs are released *via* bile fluid into the intestine and expelled from the host *via* its feces into an aquatic environment, thus completing the life cycle (Zhang et al., 1985). No commercial vaccines against *M. orientalis* are currently available, therefore, treatment of metorchiasis relies predominantly on anthelmintic treatment with praziquantel (http://www.waterpathogens.org/book/liver-flukes), hosts can be reinfected because of a lack of acquired immunity in endemic regions. Thus, new methods of controlling metorchiasis in livestock and for the treatment of drug-resistant disease in humans are urgently needed.

Recent advances in various high-throughput omics technologies has allowed for the identification of key biomolecules crucial to the processes of parasitic transmission, and the identification of novel drug and/or vaccine targets. Numerous omics data are available for socioeconomically important fluke species, such as the transcriptomes conjunction with the sequencing and assembly of their genomes of *Schistosoma japonicum*, has generated a comprehensive picture of transcriptional and genomic diversity, then combination with the omics technologies to extend large-scale screens of the transcriptome and proteome of *Schistosoma japonicum* (Liu et al., 2006; Hokke et al., 2007). The multiple omics strategy also was applied to analyses in different development stages within parasite, to elucidate host responses that mirror the stage of infection and the developmental changes that occur within the migrating parasite, it gave great hope that effective rational strategies for vaccine and drug target identification were achievable.

Systematic comparisons across parasite developmental stages and related parasites have offered insights into parasite biology, while an ‘immuno-omic approach’ has leveraged this information to allow the identification of potential vaccine and diagnostic candidates (Bennuru et al., 2016). Thus, the current study carried out a combined transcriptomic and proteomic analyses of *M. orientalis* metacercariae and adults. The resulting data will provide attractive targets for the development of new therapeutic or diagnostic interventions for controlling the development and reproduction of *M. orientalis*.

## Materials and Methods

### Parasite Samples

Metacercariae of *M. orientalis* were isolated from infected *Pseudorasbora parva* from the Wuyuer river basin (47.53°N, 124.44°E), Heilongjiang Province, China. Sheldrakes were orally infection with 100 metacercariae isolated from *P. parva* and then euthanized 6 weeks later. Adult *M. orientalis* were obtained from the liver and gallbladder of the ducks. These organs were thoroughly washed in sterile saline solution and frozen in liquid nitrogen until use. The metacercariae and adults were identified to the species level according to existing keys and descriptions (Sohn, 2009), immediately frozen in liquid nitrogen, and stored at -80°C until use.

### RNA Isolation and Illumina Sequencing

Total RNA was extracted using TRIzol reagent (Invitrogen Life Technologies, Carlsbad, CA, USA) according to the manufacturer’s protocol in three biological replicates of each *M. orientalis* development stage (pool of adults comprising of n = 50 and pool of metacercariae comprising n = 2000). Total RNA of *M. orientalis* adults or metacercariae was stored at -80°C until use. Library construction was performed according to the Illumina sample preparation for RNA-sequencing (RNA-seq) protocol. Brifely, The Oligo (dT) was used to isolate poly (A) mRNA from total RNA. Then the mRNA is fragmented into short fragments by mixing with fragmentation buffer. The cDNA was synthesized using the mRNA fragments as templates. Short fragments were purified and dissolved with EB buffer for end reparation and then connected with adapters. The suitable fragments are selected for the PCR amplification as templates. During the quality control steps, Agilent 2100 Bioanaylzer and Qubit^®^ RNA Assay Kit in Qubit^®^ 2.0 Flurometer (Life Technologies, CA, USA) were used in quantification and qualification. Sequencing of the library preparations was performed by an Illumina Hiseq X Ten platform to obtain paired-end reads.

### Transcriptome Assemble and Bioinformatic Analyses

Raw reads were subjected to quality control to obtain clean reads by removing reads with adaptors, reads containing > 10% ‘N’ residues, and low-quality reads containing > 50% bases. Clean reads were assembled into unigenes base on the default settings of the Trinity program (Grabherr et al., 2011). Unigene sequences were aligned with the NCBI non-redundant nucleotide (NT) database (Liu et al., 2016) by BLASTn (Zhang et al., 2017), and aligned with the NCBI non-redundant protein (NR) database (Liu et al., 2016), Swiss protein (Swiss-Prot) database (Liu et al., 2016), Cluster of Orthologous Groups of proteins (COG) database (Liu et al., 2016) and Kyoto Encyclopedia of genes and genomes (KEGG) database (Liu et al., 2016) by BLASTx (Zhang et al., 2017) to assign the predicted function. Hmmscan version 3.3.2 (Finn et al., 2011) was employed to match the established HMM model of protein structure domain among the Pfam database (Zhang et al., 2017). ESTScan version 3.0.3 (Iseli et al., 1999) was employed to predict protein coding sequences (CDS) with default setting. Blast2GO (Götz et al., 2008) was employed to classify unigenes to Interpro and Gene Ontology (GO) terms including molecular function, biological processes, and cellular components (Conesa et al., 2005) and analyzed the distribution of *M. orientalis* gene functions at the macro-level. The clean reads were deposited in the Sequence Read Archive database of NCBI (accession no. PRJNA474572), with sra run accessions numbers SRR7410653 and SRR7410652 for adult and metacercaria *M. orientalis*, respectively. The assembled cDNA sequences were deposited in the Transcriptome Shotgun Assembly (TSA) database of GenBank (accession no. GGVK00000000).

### Identification of Genes Differentially Expressed Between Adult and Metacercariae *M. orientalis*

Unigene expression was calculated based on the Fragments Per kb per Million reads (FPKM) method (Mortazavi et al., 2008). The FPKM values were used directly used to compare the differences in gene expression levels between the two developmental stages. The Benjamini-Hochberg procedure is used to perform multiple corrections to *p*-values and generate false discovery rate (FDR) values. Differentially expressed genes (DEGs) were identified with an adjusted FDR < 0.005 found by DESeq2 version 1.34.0 (Love et al., 2014).

### Quantitative Real-Time PCR Validation

Partial of total RNA same as transcriptome sequencing were used for quantitative real-time PCR (qRT-PCR) validation. Primers designed according to the Illumina sequencing data are listed in **Table S1**. CDNA was synthesized from total RNA using the reverse transcription kit (Takara, Dalian, China) following the manufacturer’s instructions. Thermocycling conditions were: 40 cycles each with 95°C for 10 s for denaturation, 60°C for 20 s for annealing, and 72°C for 30 s for extension, performed in StepOne Plus Real-Time PCR System (Applied Biosystems, Foster City, CA, USA) with SYBR Green Pre-mix Ex Taq (Takara, Dalian, China) in triplicate. Relative gene expression was calculated using the 2^-ΔΔCt^ method with β-actin (GenBank no. EU109284) as the internal control. The correlation coefficients between the transcriptome and qRT-PCR values were calculated.

### Protein Preparation and Digestion

Each *M. orientalis* development stage of total proteins (pool of adults comprising of n = 50 and pool of metacercariae comprising n = 2000) were extracted using protein lysis buffer (7 M urea, pH 8.0) in three biological replicates, and were lysed by sonication on ice (2/3 s, 5 min) using a high-intensity ultrasonic processor (Scientz Biotechnology Co. LTD, Ningbo, China).

The lysate was centrifuged at 20,000 × g for 20 min at 4°C in order to remove debris. After centrifugation, the supernatant was treated with 10 mM dithiothreitol for 60 min at 37°C. Then, the samples were alkylated with 55 mM iodoacetamide, protected from light for 45 min at room temperature. The concentration of protein was quantified using the BCA protein assay kit (Pierce, Rockford, IL, USA). For each sample approximately 10 μg of protein was subjected to 12.5% sodium dodecyl sulfate-polyacrylamide gel electrophoresis (SDS-PAGE) to assess protein integrity.

The protein samples for 50 μg were diluted with 30 mM HEPES until the concentration of urea becomes < 2 M. Trypsin was added into each sample at an enzyme to protein ratio of 1:50 and the samples were further digested overnight at 37°C. Enzymatic digestion was terminated by adding 0.5% (v/v) formic acid. Finally, peptides of each sample were desalted and concentrated using Sep-Pack C_18_ Cartridges (Waters, Worcester, MA, USA).

### HPLC and LC-MS/MS

All samples filtering experiment were separated by an HPLC system (Easy-nanoLC, Thermo Scientific, Chelmsford, MA, USA) connected to an orbitrap fusion mass spectrometer (Thermo Scientific, Chelmsford, MA, USA). Peptides were resuspended with phase A [2% acetonitrile (ACN), 0.1% formic acid (FA)] and centrifuged at 12,000× *g* for 10 min. The supernatant was loaded on the trap column to be enriched and desalted. Then, the peptides were separated at a flow rate of 300 nL/min on a 15 cm analytical column (Beijing Qinglian Biotech, China, 150 μm ×150 mm, 100A˚, 1.9 μm) connected to the trap column. The linear gradient of LC was set at 3% buffer B (95% ACN, 0.1% FA) (from 0 to 5 min), 8–28% buffer B (from 5 to 107 min), and finally a hold at 28–80% buffer B (from 107 to 120 min).

Peptides were ionized by a nano-electrospray ion source and then identified by the orbitrap fusion mass spectrometer (Thermal Scientific, Chelmsford, MA, USA) in the mode of DDA (data-dependent acquisition). The scan of first-grade MS ranged from 350 to 1550 m/z at a resolution of 120,000 and an automatic gain control (AGC) target of 2×10^6^. The scan of second-grade MS was initiated as 100 m/z at a resolution of 30,000 with a dynamic exclusive time of 30 ms and an AGC target of 5 × 10^4^. The mode of second-grade MS spectra was high-energy collisional dissociation, and inject ions for all available parallelizable time.

### Protein Quantification and Bioinformatic Analyses

Raw mass spectra were searched against *M. orientalis* transcriptome database in present study, and protein identification was performed using MaxQuant version 1.5.3.30 (Wiśniewski et al., 2009). The search parameters were set as follows: first and main search peptide tolerances of 20 ppm and 6 ppm, respectively, precursor ion mass tolerances of 20 ppm, a maximum of two missed trypsin cleavage sites, fixed cysteine carbamidomethylation, and variable methionine oxidation. Then, the acetylated sites were filtrated at the level of site decoy fraction ≤ 1% to obtain the significant modification. The *p*-value for identification and quantification of proteins was set as *p* ≤ 0.05 and acetylated proteins with a fold-change of two were deemed as differentially expressed proteins (DEPs). Protein annotation used BLASTP on the UniProt database (Wang et al., 2011) with default parameters. GO enrichment analysis was used to determine whether the identified proteins were enriched in certain functional groups, as compared with the uniprot *Clonorchis sinensis* dataset (Wang et al., 2011), and Fisher’s exact test was used for the analysis. Next, the identified proteins were blasted against the kyoto encyclopedia of genes and genomes (KEGG) (Wang et al., 2011) for orthology identification of the corresponding genes, and subsequently mapped to metabolic and regulatory pathways in KEGG. The proteomic data were deposited in the iProX platform (http://www.iprox.org) with the project no. IPX0003502000.

### Parallel Reaction Monitoring Validation

Partial of total proteins same as proteome sequencing were used for further targeted quantification by Parallel Reaction Monitoring (PRM) by Beijing QLBio Biotechnology Co. (China). Briefly, An AQUA stable-isotope peptide as an internal standard reference was spiked in with each sample. Digested peptides were desalted on C_18_ stage tips prior to reversed phase chromatography on an Easy-nanoLC system (Thermal Scientific, Chelmsford, MA, USA). One hour liquid chromatography at a flow rate of 300 nL/min was used with the following gradients: 3 to 28% buffer B in 107min and 28 to 80% buffer B in 3 min. PRM analyses was performed using an orbitrap fusion mass spectrometer (Thermal Scientific, Chelmsford, MA, USA). Optimal collision energy, charge state, and retention time for the most significantly regulated peptides were generated experimentally using unique peptides of high intensity and confidence for each target protein. The mass spectrometer was operated in position ion mode with the following parameters: the full scan was collected with a resolution of 120,000 at 200m/z, the AGC target was 2×10^6^ and the maximum injection time was at 100 ms. All PRM data analyses and data integration were performed using Skyline version 3.5.0 (MacLean et al., 2010). Three replicates were included for each sample in the PRM-MS analyses. Relative peptide quantification was calculated by dividing the peptide peak area. A two-tailed Student’s t-test was used to estimate the significance of the difference in relative peptide abundance between *M. orientalis* adults and metacercariae.

### Conjoint Analyses of Transcriptome and Proteome

In order to examine the detail post-translational regulation between transcriptome and proteome in adult and metacercaria *M. orientalis*, the fold changes of mRNA and protein were compared. In briefly, the fold changes were got in mRNA and protein level separately between adult stage and metacercariae-stage *M. orientalis*. And then, fold change ratio was calculated (As the following formula).


$$\text{Fold change ratio}=\frac{\text{Fold change}(\text{Protein})}{\text{Fold change}(\text{mRNA})}$$

We propose for most genes, the fold changes of mRNA and protein are similar. In order to get those genes with significant different fold change, significance A were calculate using MaxQuant version 1.5.3.30 (Wiśniewski et al., 2009), and those genes with the p value less than 0.05 and fold change ratio greater than 2 or less than 2 were consider as the significance up or down genes.

The statistical analyses involved in this study was implemented on R 4.0.3 platform (https://www.r-project.org/). Visualization of graphics were built in GraphPad Prism 8.0 (GraphPad Software, La Jolla, CA, USA) and R environment using the ggplot2 version 3.3.5 and ggpubr version 0.4.0 by online website https://github.com/kassambara/ggpubr/.

## Results

### Overview of Transcriptomic Analyses and Quantitative Proteomics

**Figure 1:**
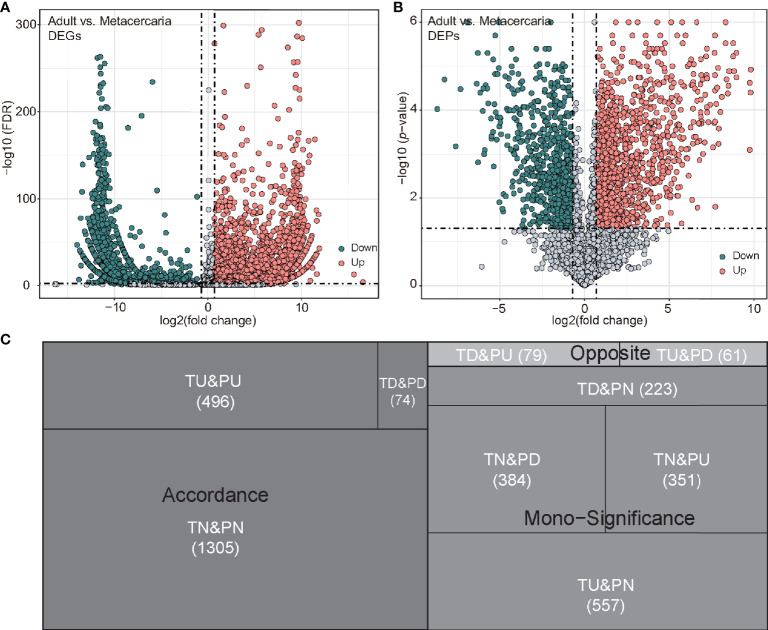
Differentially expressed genes (DEGs) and differentially expressed proteins (DEPs) between adult stage and metacercariae stage. **(A, B)** presented DEGs and DEPs volcanoes, respectively. The differential expression cut-off of mRNA was FDR < 0.005 and 2-fold change, while the differential expression cut-off of protein was *p*-value <0.05 and 2-fold change. **(C)** The tree diagram shows the results of mRNA and protein co-expression. TU, TD and TN represent up-regulated, down-regulated and unchanged mRNA, respectively. PU, PD and PN represent up-regulated, down-regulated and unchanged protein, respectively.

A total of 47,396,124 and 51,014,100 clean reads were obtained from *M. orientalis* adults and metacercariae, respectively. The average ratio of clean reads to raw reads was 94.43% (**Table S2**). A total of 254,543 (>200 nt) transcripts were produced by the Trinity program for all samples. The N50 size was 1,002 nucleotide base pairs, which is shorter than other helminth transcriptomes (Liu et al., 2016; Zhang et al., 2017). Removing the redundancy resulted in 100,234 unigenes based on at least 0.3 FPKM in all samples. Functional annotation of the 100,234 unigenes data set was carried out using seven in public databases (Nr, Nt, Pfam, COG, Swiss-Prot, KEGG, and GO), resulting in 58.27% (n = 58,402) of the data set being annotated in at least one database (**Table 1**). Using a cut-off FDR of < 0.005 and a twofold change identified 13,823 DEGs in adult versus metacercariae, of which 4,773 were upregulated and 9,050 were downregulated (**Table S3**; **Figure 1A**).

**Table 1:** Bioinformatics annotation of transcriptome unigenes and proteome proteins.

Bioinformatics annotations of unigenes	Number of Unigenes	Percentage (%)	Number of Protein	Percentage (%)
Annotated in NR	52,144	52.02	–	–
Annotated in NT	25,397	25.34	–	–
Annotated in KO	16,834	16.79	1,623	45.98
Annotated in SwissProt	30,959	30.89	–	–
Annotated in PFAM	35,949	35.87	–	–
Annotated in GO	36,042	35.96	2,385	67.56
Annotated in KOG	20,732	20.68	–	–
Annotated in all Databases	3,163	3.16	–	–
Annotated in at least one Database	58,401	58.26	–	–
Total Unigenes/Proteins	100,234	100	3,530	100

In total, 21,604 peptides, 10,704 unique peptides, and 3,530 proteins were determined *via* proteomic analyses (**Table S4**). In terms of protein mass distribution, most proteins (73.5%) had molecular weights ranging from 10 to 70 ku, 12.1% of proteins had a molecular weight > 100 ku. Using KEGG and GO database annotations, 45.98% of proteins (1,623/3,530) were annotated to 1,036 GO terms and 67.56% proteins (2,385/3,530) were annotated to 1,140 KEGG pathways (**Table 1**). Using a twofold change and *p*-value < 0.05 as a threshold resulted in 1,445 DEPs detected by the proteomic analyses (adult versus metacercariae), among which 519 proteins were up-regulated and 926 proteins were down-regulated (**Table S5**; **Figure 1B**).

By comparing the RNA-seq data with the proteomic data, all proteins were matched to the corresponding unigenes. Of these, 2,225 genes, including 780 DEGs and 1445 DEPs displayed differential expression at either the mRNA or protein levels between adult stage and metacercariae-stage *M. orientalis*. Of these, 496 genes were consistently upregulated between the adult stage and metacercariae stage, and 74 genes were consistently downregulated between the adult stage and metacercariae stage (**Table 2**; **Figure 1C**). However, 1,655 genes showed inconsistent expression at the mRNA and protein levels between the adult stage and metacercariae stage, which might result from post-translational regulation or modifications.

**Table 2:** Combined analyses of transcriptome and proteome data.

Classification	Transcriptomic/Proteomic	Adult versus Metacercariae
Accordance	Invariant/Invariant	1305
	Up/Up	496
	Down/Down	74
Mono-Significance	Down/Invariant	223
	Up/Invariant	557
	Invariant/Down	384
	Invariant/Up	351
Opposite	Up/Down	61
	Down/Up	79

### Transcriptomic Analyses of Adult and Metacercariae Stages of *M. orientalis*

**Figure 2:**
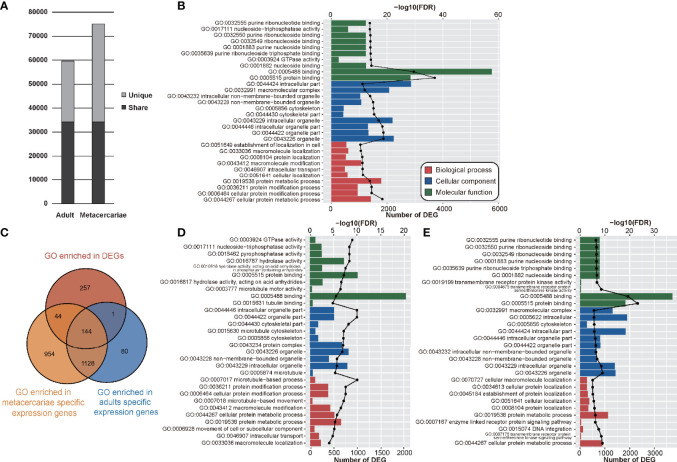
The results of transcriptome analyses between adult stage and metacercariae stage. **(A)** The bar chart shows the number and sharing of genes expressed in adult stage and metacercariae stage. **(B)** Demonstrated the gene ontology (GO) assigned to the DEGs. **(C)** Demonstrated the relationship between the enrichment function and stage-specific genes. **(D)** Enrichment of up-regulated and down-regulated DEGs (adult versus metacercariae) in GO function. **(E)** Enrichment of down-regulated DEGs (adult vs metacercariae) in GO function.

Analyses of gene sharing between the adult stage and metacercariae-stage *M. orientalis* revealed 34,228 unigenes to be co-expressed by adults and metacercariae, whereas most (n = 66,006) exhibited stage-specific expression (adult = 25,426; metacercariae = 40,850) (**Figure 2A**). This suggested that the transcriptome level during the metacercariae stage is high, which might be related to the infection ability of metacercariae. There were 1,272 terms common to both adults and metacercariae. Only 81 GO terms were unique to the adults, compared with 998 GO terms in the metacercariae. The unique GO terms in the adults included GO:0044163, GO:0075521, and GO:0071479, involving host cytoskeleton, microtubule-dependent intracellular transport of viral material towards nucleus, and cellular response to ionizing radiation. Specific GO entries in the metacercariae included GO:0004664, GO:0004298, GO:0005839, and GO:0051603. These functions involved prephenate dehydratase activity, threonine-type endopeptidase activity, proteasome core complex, and proteolysis involved in cellular protein catabolism.

GO analyses were performed on DEGs and all DEGs were assigned to 4,389 GO terms, of which 446 GO terms were significantly enriched (*p* < 0.05). We focused on these enriched functions. In terms of biological processes, 267 GO terms were associated with these DEGs, most of which were involved in cellular protein metabolic process, cellular protein modification process, protein modification process. In terms of the cellular component, 77 GO terms were associated with these DEGs and were mainly involved in organelle, organelle part, and intracellular organelle part. By contrast, 102 molecular function GO terms were associated with these DEGs, including protein binding, binding, nucleoside binding, GTPase activity, and purine ribonucleoside triphosphate binding (**Figure 2B**; **Table S6**). In addition, nucleocytoplasmic transport (GO:0006913) was the only function that was significantly enriched among the genes unique to adults. By contrast, 44 GO terms were significantly enriched that involved genes unique to metacercariae (**Figure 2C**).

The up- and down-regulated genes were further analyzed to better understand the DEGs between adult and metacercariae-stage *M. orientalis*. All the upregulated genes were assigned to 139 GO terms (**Table S7**). Among these, up-regulated genes were uniquely enriched in 64 GO functions, mainly related to microtubules, and were significantly enriched in functions such as microtubule-based processes, microtubule-based movement, microtubule motor activity, and tubulin binding (**Figure 2D**). In addition, numerous up-regulated genes were enriched in GO functions of catalytic activity, hydrolase activity, and catalytic complexes. By contrast, down-regulated genes were assigned to 234 GO terms, of which 159 were uniquely enriched, including transmembrane receptor protein serine/threonine kinase activity, transmembrane receptor protein serine/threonine kinase signaling pathway, DNA integration, transmembrane receptor protein kinase activity, and enzyme linked receptor protein signaling pathway (**Figure 2E**). In addition, numerous down-regulated genes were concentrated in primary metabolic processes, cellular metabolic processes, macromolecule metabolic processes, and cellular macromolecule metabolic processes. Interestingly, a few genes participated in a variety of negatively regulated functions, such as neuropeptide hormone activity, negative regulation of proteasomal protein catabolic processes, and negative regulation of mitotic nuclear division.

**Figure 3:**
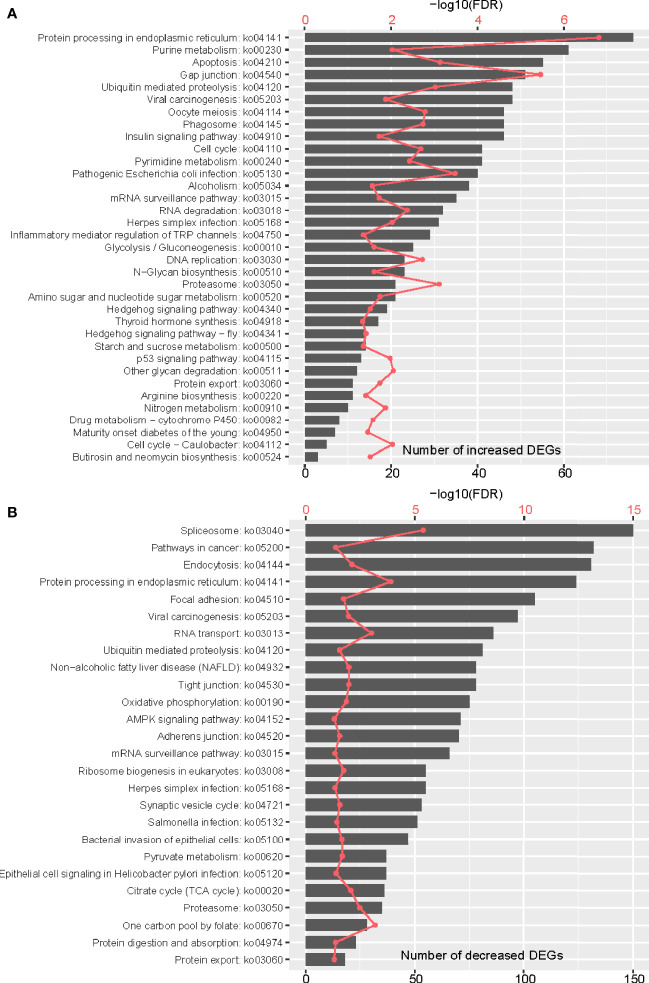
Functional analyses of DEGs between adult stage and metacercariae stage. **(A)** showed the enrichment of DEGs in GO function, and **(B)** showed the enrichment of DEGs in KEGG function. Only results with FDR < 0.05 are shown.

KEGG pathway enrichment analyses of DEGs showed that 215 up-regulated DEGs were significantly enriched in 35 pathways (*p <*0.05) (**Figure 3A**; **Table S8**). Consistent with the GO analyses, up-regulated genes were enriched in a variety of pathways related to reproduction, substance metabolism, and biosynthesis, such as oocyte meiosis (ko04114), glycolysis/gluconeogenesis (ko00010), pyrimidine metabolism (ko00240), nitrogen metabolism (ko00910), N-glycan biosynthesis (ko00510), purine metabolism (ko00230), starch and sucrose metabolism (ko00500) and arginine biosynthesis (ko00220). Similarly, various up-regulated genes participated in pathways related to genetic information processing, suggesting vigorous reproductive behavior during the adult stage. Of note, up-regulated genes were significantly enriched in the ko05130 pathway, which is mainly involved in the encoding of ACTB_G1 (Actin beta/Gamma 1), a protein with a key role in adult motion. In addition, these genes were enriched in the p53 signaling pathway (ko04115), which is highly associated with cancers and mainly regulates the apoptosis and senescence of cells. Interestingly, genes that were highly expressed in adults were also involved in butirosin and neomycin biosynthesis (ko00524), thyroid hormone synthesis (ko04918), inflammatory mediator regulation of TRP channels (ko04750), and drug metabolism - cytochrome P450 (ko00982). These findings could provide clues to the mechanisms by which the parasites fight bacteria and immune evasion.

Of the down-regulated DEGs, 261 were significantly enriched in 26 pathways (*p* < 0.05) (**Figure 3B**). Most of these genes expressed in metacercariae were involved in various biological cycles and some metabolic processes, such as the citrate cycle (TCA cycle) (ko00020), oxidative phosphorylation (ko00190), pyruvate metabolism (ko00620), and one-carbon pool by folate (ko00670). In addition, many hyperexpressed genes in metacercariae were enriched in the AMPK signaling pathway (ko04152), a fuel sensor and regulator that promotes ATP-producing and inhibits ATP-consuming pathways in various tissues. This suggests that the parasite regulates its energy use during the metacercariae stage when food intake is not possible. Interestingly, the overexpressed metacercariae genes were also involved in a variety of disease-related pathways, such as herpes simplex infection (ko05168), epithelial cell signaling in *Helicobacter pylori* infection (ko05120), bacterial invasion of epithelial cells (ko05100), non-alcoholic fatty liver disease (NAFLD) (ko04932), viral carcinogenesis (ko05203), and salmonella infection (ko05132). These findings could provide clues to a potential association between the *M. orientalis* metacercariae and these diseases.

### Validation of RNA-Seq Profiles by qRT-PCR

To validate the transcriptome data, eight genes (three up-regulated and five down-regulated in adult stage versus metacercariae stage) were selected randomly among the DEGs, and their expression levels were verified by qRT-PCR (**Figure S1**). The results showed that DEGs of phosphoglycerate mutase, translation initiation factor 3 subunit B, and cathepsin F precursor were up-regulated in the adult stage versus metacercariae stage, whereas cytoplasmic 1, serpin, glutamine synthetase, elongation factor 1-gamma, and lactate dehydrogenase were up-regulated in metacercariae stage versus adult *M. orientalis*, which showed a similar expression trend to the transcriptome analysis, providing evidence on the reliability of the transcriptome sequencing results.

### Proteomic Analyses of Adult and Metacercariae-Stage *M. orientalis*

**Figure 4:**
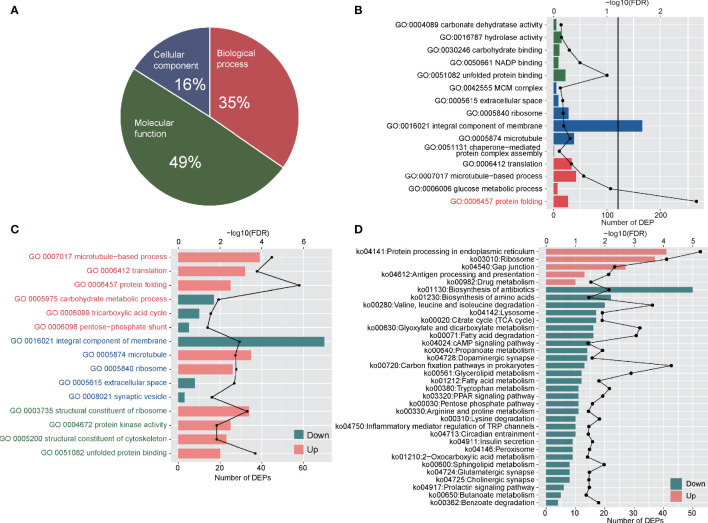
Functional analyses of DEPs between adult stage and metacercariae stage. **(A)** Demonstrated the classification of DEPs by cellular component, biological process and molecular function. **(B)** The DEPs enriched in GO function. The red font indicates the significant enrichment function. **(C)** The increased and decreased DEPs enriched in GO function. The red font represents biological process classification, the blue font represents cellular component classification, and the green font represents molecular function classification. **(D)** The up-regulated DEPs and down-regulated DEPs enriched pathways in KEGG function. The discount graph represents the -log10 value of FDR and only shows the results of FDR < 0.05.

GO analyses were performed on DEPs, and all DEPs were assigned to 630 GO functions. Among these, there were 218, 101, and 311 GO terms for biological processes, cellular components and molecular functions, respectively (**Figure 4A**). Enrichment analyses showed that only protein folding (GO:0006457) was significantly enriched (FDR < 0.05) (**Figure 4B**; **Table S9**). The DEPs up- and down-regulated between the adult and metacercariae *M. orientalis* were then analyzed. In terms of biological processes, up-regulated proteins were significantly enriched in protein folding, microtubule-based processes and translation, whereas down-regulated proteins were significantly enriched in carbohydrate metabolic processes, TCA cycle, and the pentose-phosphate shunt (**Figure 4C**). In terms of cellular components, up-regulated proteins were significantly enriched in microtubules and ribosomes, whereas down-regulated proteins were significantly enriched in extracellular space, integral membrane components, and synaptic vesicles. In terms of molecular functions, up-regulated proteins were significantly concentrated in the unfold protein binding, structural dynamics of ribosomes, protein kinase activity, and structural constituents of cytoskeleton categories, whereas down-regulated proteins were assigned to 205 GO terms (**Figure 4C**), however, no protein were enriched for any function.

KEGG pathway enrichment analyses were performed for DEPs, and all DEPs were assigned to 335 KEGG pathways (**Table S10**). These proteins were significantly enriched in glutathione metabolism (ko00480) and glycerolipid metabolism (ko00561). Up-regulated and down-regulated proteins enrichment analyses showed that a total of 388 up-regulated DEPs were involved in 294 KEGG pathways, and 265 down-regulated DEPs were involved in 304 pathways. The up-regulated proteins were significantly enriched in protein processing in endoplasmic reticulum, ribosomes, gap junctions, antigen processing and presentation, and drug metabolism (**Figure 4D**). By contrast, the down-regulated proteins were significantly enriched in 29 pathways, among which numerous proteins were enriched in the biosynthesis of antibiotics, biosynthesis of amino acids, valine, leucine and isoleucine degradation pathways (**Figure 4D**). In addition, a few down-regulated proteins were enriched in inflammatory mediator regulation of TRP channels, PPAR signaling pathways, and cAMP signaling pathways.

### Verification of DEPs by PRM

PRM is an ion monitoring technology based on high resolution and high precision mass spectrometry, and can selectively detect target proteins and peptides, such as post-translational modifications peptides. To confirm DEPs in label-free analyses between adult stage and metacercariae-stage *M. orientalis*, eight significant DEPs were selected randomly for PRM analyses. The results showed the similar expression trends for the label-free proteomics data and PRM data (**Table 3**), verifying the accuracy and reliability of the proteome analyses.

**Table 3:** Confirmation of DEPs detected in label-free analyses using PRM assay.

Accession	Description	Labelfree	PRM
		Ratio (Ma versus Mm)	Ratio (Ma versus Mm)
Cluster-2852.76449.orf1	saposin	0.42	0.43
Cluster-2852.68591.orf1	tegument antigen	0.78	0.43
Cluster-2852.68242.orf1	cathepsin F	118.36	55.80
Cluster-2852.85169.orf1	glutathione transferase	102.19	51.20
Cluster-2852.88752.orf1	acetylcholinesterase	0.02	0.09
Cluster-2852.60070.orf1	major egg antigen	50.98	10.94
Cluster-2852.68699.orf1	protein kinase A	46.96	10.15
Cluster-2852.53609.orf1	calreticulin	35.27	11.93

### Combined Transcriptome and Proteome Functional Analyses

**Figure 5:**
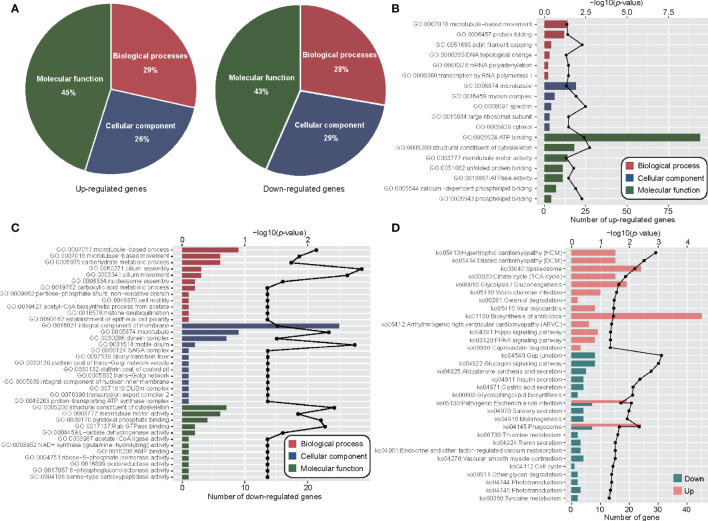
Functional analyses combined transcriptome and proteome. **(A)** The pie plot shows the classification of co-up-regulated and co-down-regulated mRNA and proteins in GO function. **(B)** Co-up-regulated gene enrichment of GO function. **(C)** Co-down-regulated gene enriched GO function. **(D)** KEGG enriched function of genes with consistent mRNA and protein expression levels. The discount graph represents the -log10 value of *p*-value and only shows the results of *p*-value < 0.05.

In order to examine the detail post-translational regulation between transcriptome and proteome in adult and metacercaria *M. orientalis*, the significance up or down genes were calculated by significance A method (**Table S11**). Further analyses of the significance up or down genes were conducted through GO and KEGG pathways. Analyses of potential translation regulation were performed to obtain possible up-regulation and down-regulation results. Among 669 up-regulated genes, 230, 213, and 364 genes were assigned to biological processes, cellular components and molecular functions, respectively (**Figure 5A**; **Table S12**). These up-regulated genes were associated with actin filament capping, spectrin, glucose metabolic processes, arginine metabolic processes, structural constituents of cytoskeleton, myosin complexes and ATP binding (**Figure 5B**). In addition, some up-regulated genes were enriched in urea cycle, glucose metabolic processes, aromatic amino acid family metabolic processes, TCA cycle, and glycolytic processes (**Table S13**). This suggested that energy metabolism activities increased during metacercariae development. In addition, these up-regulated genes were also involved in the phagosome, spliceosome, and PPAR signaling and Hippo signaling pathways. Interestingly, many of the up-regulated genes were also involved in a variety of disease processes, such as hypertrophic cardiomyopathy (HCM), dilated cardiomyopathy (DCM), *Vibrio cholerae* infection, and viral myocarditis. Among 235 down-regulated genes, 53, 55, and 83 genes were assigned to biological processes, cellular components, and molecular functions, respectively. These decreased genes were associated with cilium assembly and movement, motile cilia, microtubule pyridoxal phosphate binding, and Rab GTPase binding (**Figure 5C**). Some down-regulated genes were also enriched in gap junctions, glucagon signaling pathway, aldosterone synthesis and secretion, insulin secretion, and other pathways (**Figure 5D**). These results suggest that although gene and protein expression levels were not always consistent, there was a high degree of consistency between the functions of DEGs and DEPs in the different stages of *M. orientalis*.

## Discussion

The liver fluke *M. orientalis* is an economically important pathogen of livestock worldwide, as well as being an important neglected zoonosis. Metorchiasis control is reliant on the use of drugs, particularly praziquantel, which is effective against multiple parasite stages (http://www.waterpathogens.org/book/liver-flukes). However, the spread of parasites resistant to praziquantel has intensified the pursuit for novel control strategies (Fairweather et al., 2020). Emerging omic technologies are helping advance our understanding of parasite biology, specifically the molecules that act at the host-parasite interface and are central to infection, virulence and long-term survival within the host (Prasopdee et al., 2019). To better understand the biology of *M. orientalis*, transcripts from the adult stage were sequenced by next-generation sequencing in a previous study (Gao et al., 2018). Although this published data set provided significant insights into the transcriptome of *M. orientalis*, only the adult stage was represented, and this initial study performed a qualitative exploration of the transcriptome, quantitative assessment of transcription during the life-cycle of this parasite was not possible at the time of study. To overcome these limitations for *M. orientalis*, a next-generation sequencing platform and proteomics were used to develop a global view of the transcriptomes of adult and metacercariae stages of *M. orientalis* in the present study.

In total, 13,823 distinct genes and 1,445 proteins were found to be differentially expressed between adult and metacercariae *M. orientalis*, which is significantly higher than that found for *C.sinensis, O. felineus*, *and O. viverrini* (Jex et al., 2012; Huang et al., 2013; Pomaznoy et al., 2016). Although some genes involved in basal and energy metabolisms were abundantly expressed in both stages of *M. orientalis*, some genes showed differential expression because of the different biological characteristics of the two developmental stages. Adult worms produce abundant eggs daily, thus the transcriptome profile is tightly linked with egg production. In the current study, many reproduction-associated proteins, such as vitelline B precursor protein and egg protein, were highly expressed at both the transcription and translation levels in the adult stage. Tyrosinase has a key role in the formation of eggshell, which originates from the vitelline cells inside the vitellaria. In the current study, tyrosinase was highly expressed in the adult stage, a result that was consistent with a previous report (Anderson et al., 2015). In addition, because adults *M. orientalis* inhabit the bile duct of their definitive host, and biliary duct cells are frequently exposed to liver-derived endogenous and exogenous toxins, carcinogens, drugs, and their metabolites (xenobiotics), the flukes have evolved an antioxidant system to protects its cells against such compounds. For example, glutathione-S-transferases can protect the parasite by reducing lipid hydroperoxides, as well as detoxifying xenobiotic substrates *via* glutathione conjugation.

In metacercariae stage, because the parasite remains dormant and maintains a low metabolic ratee, some ribosomal proteins, such as elongation factor 2 and structural housekeeping genes, such as Heat shock proteins 70, were transcribed at a higher rate in this stage. Such structural housekeeping genes can maintain the most basic life characteristics and consume the least amount of energy until it is engulfed by its definitive host (Pomaznoy et al., 2016). The metacercariae experience a significant thermal change when they move from their intermediate host (freshwater fish) to the stomach of the definitive host (poultry or mammals), thus, the high transcription of the heat shock proteins might be related to their response to thermal-induced stresses (Abou-El-Naga, 2020). Moreover, cysteine protease was also highly transcribed in metacercariae, and is an essential enzyme involved in initiating excystation (Yoo et al., 2011).

*C. sinensis* and *O. viverrini* have been assessed as carcinogenic biological agents to humans by the Agency for Research on Cancer, and *O. felineus* has also been reported to be associated with the development of cholangiocarcinoma by comparing previously reported molecular targets (Pomaznoy et al., 2016; Prueksapanich et al., 2018). However, as a member of the Opisthorchiidae, little is known about the carcinogenicity of *M. orientalis*. The pathways of pathogenesis of opisthorchiasis-associated cholangiocarcinoma are thought to be multifactorial, including mechanical damage, inflammation-induced immunopathology, and direct effects of fluke-secreted growth factors (Pakharukova and Mordvinov, 2016). Cathepsin F and granulins are considered as the crucial carcinogenic factors secreted by flukes (Pomaznoy et al., 2016). Cathepsin F is a cysteine protease family, which can induce inflammation and promote malignancy. The fluke secretes several cathepsin F cysteine proteases into the bile duct that could induce or contribute to the pathologies associated with hepatobiliary abnormalities (Pinlaor et al., 2009). Granulins are growth factors that can be secreted into the bile ducts, they have mitogenic properties that drive cell proliferation, creating a tumorigenic environment (Arunsan et al., 2020). In the present study, cathepsin F and granulins were found most highly expressed in *M. orientalis* (mainly in the adult stage), which is consistent with previous reports (Pomaznoy et al., 2016; Young et al., 2021). This suggests that *M. orientalis* is a neglected trematode with potential carcinogenic implications, although further research is needed.

Based on the KEGG pathway, the most enriched pathway terms between adult stage and metacercariae *M. orientalis* included protein processing in endoplasmic reticulum (200 genes; 45 proteins), spliceosome (200 genes; 15 proteins), and proteasome (56 genes; 5 proteins), indicating the involvement of active metabolic processes in the development of *M. orientalis* metacercariae to adults. Interestingly, signaling pathways associated with “liver fibrosis” were also identified, namely the TGF-β signaling pathway (44 genes; two proteins). Similar to *O. felineus* and *C. sinensis*, *M. orientalis* can caused liver fibrosis during chronic infection (Wang et al., 2020). Previous studies showed that liver fibrosis was orchestrated by a complex network of signaling pathways involved in regulating the deposition of extracellular matrix (Yan et al., 2015). Among these signaling pathways, the TGF-β signaling pathway has been shown to have an important role in the development of liver fibrosis caused by parasitic infection (Gao et al., 2018). TGF-β is a major pro-fibrotic cytokine, with a crucial role in orchestrating fibrogenesis. TGF-β1 triggers its downstream signaling pathway meditated by TGF-β type I (TGFβRI) and type II receptors (TGFβRII), causing Smad2 and Smad3 phosphorylation. Phosphorylated Smad2 and Smad3 rapidly combine with Smad4 and subsequently migrates to the nucleus, leading to mass of fibrotic genes expression (Hata and Chen, 2016; Hu et al., 2018). Meanwile, the TGF-β signaling pathway has also been related to egg-laying behavior in *Fasciola gigantica* (Zhang et al., 2017). Thus, these data provide attractive targets for the development of new therapeutic or diagnostic interventions for controlling the development and the reproductive process of *M. orientalis*.

## Conclusion

The present study revealed a transcriptome and proteome data set for adult stage and metacercariae-stage *M. orientalis* that significant expands the currently gene repertoire of this parasitic trematode. The characterization of these transcriptome and proteome data has implications for an improved understanding of the biology of *M. orientalis*, and will facilitate the development of intervention agents for this and other pathogenic flukes of human and animal health significance.

## Data Availability Statement

The datasets presented in this study can be found in online repositories. The names of the repository/repositories and accession number(s) can be found in the article/**Supplementary Material**.

## Ethics Statement

The animal study was reviewed and approved by the National Institute of Animal Health Animal Care and Use Committee of the Heilongjiang Bayi Agricultural University.

## Author Contributions

C-RW designed the project and experiments. J-FG analyzed the data and writing original manuscript. Q-BL writing, reviewing and editing manuscript. R-FM and Y-YS validation the data. Y-YC and Y-YQ conducted the experiments. Q-CC analyses and interpretation of data. All authors contributed to the article and approved the submitted version.

## Funding

This work was supported by National Natural Science Foundation of China (31972703; 32172886), Natural Science Foundation of Heilongjiang Province (LH2021C071), Heilongjiang Provincial Postdoctoral Science Foundation (LBH-Z19191) and Heilongjiang Bayi Agricultural University Support Program for San Heng San Zong (TDJH202002).

## Conflict of Interest

The authors declare that the research was conducted in the absence of any commercial or financial relationships that could be construed as a potential conflict of interest.

## Publisher’s Note

All claims expressed in this article are solely those of the authors and do not necessarily represent those of their affiliated organizations, or those of the publisher, the editors and the reviewers. Any product that may be evaluated in this article, or claim that may be made by its manufacturer, is not guaranteed or endorsed by the publisher.

## Supplementary Material

Supplementary Figure 1 Validation of RNA-seq profiles by quantitative real-time PCR.
