# Supplementary figures and images for: Integrative Transcriptomics and Proteomics Analyses to Reveal the Developmental Regulation of Metorchis orientalis: A Neglected Trematode With Potential Carcinogenic Implications
Source: Front Cell Infect Microbiol. 2021 Dec 2;11:783662. doi: 10.3389/fcimb.2021.783662 (PMC8674872; doi:10.3389/fcimb.2021.783662)

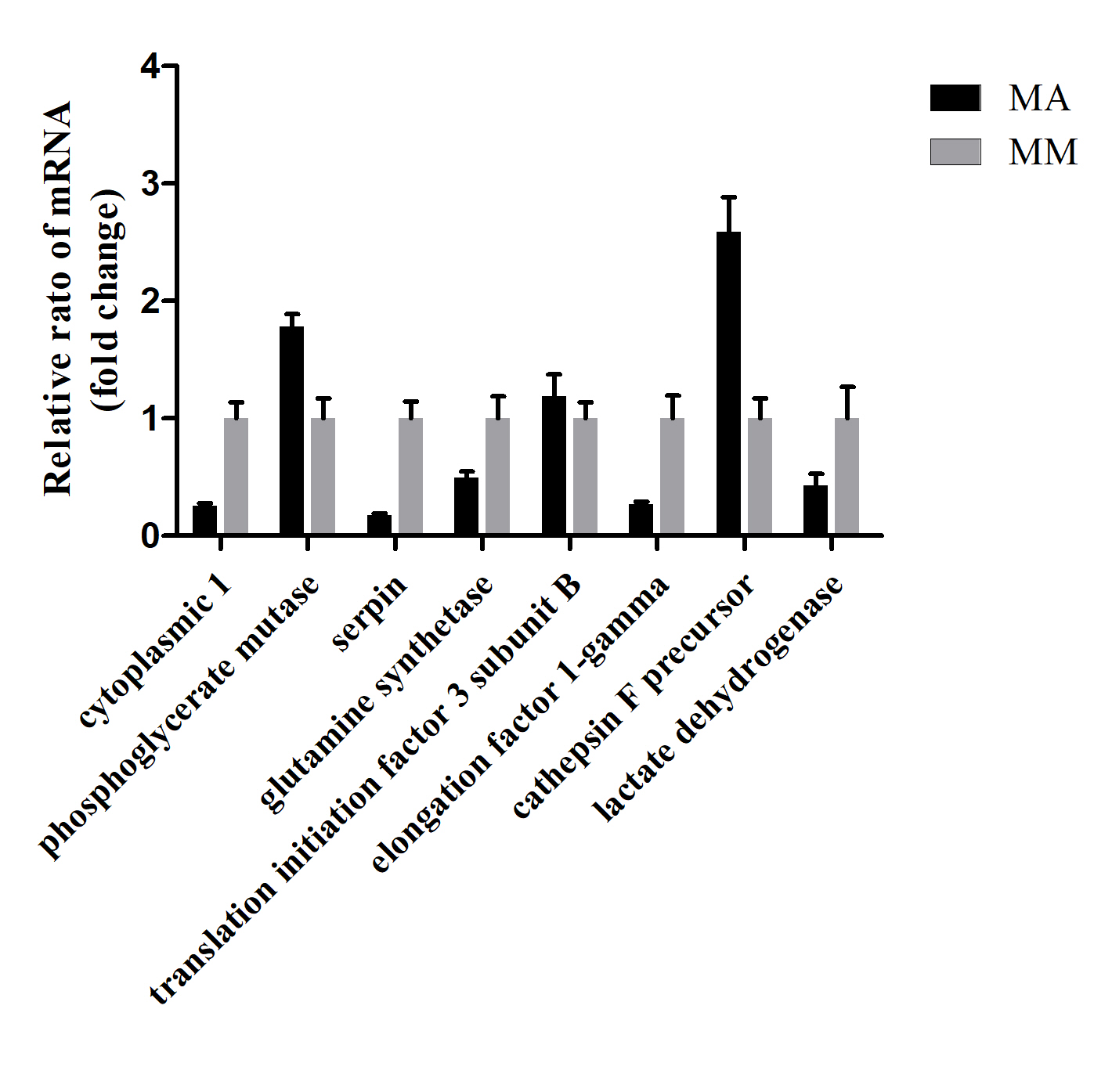

Supplement: Supplementary Figure 1 — Validation of RNA-seq profiles by quantitative real-time PCR. [file Image_1.jpg]
